# Low‐Molecular‐Weight *Angelica sinensis* Polysaccharide Improves Neurological Function and Neuroplasticity Following Cerebral Ischemia–Reperfusion Injury

**DOI:** 10.1002/fsn3.71010

**Published:** 2025-09-26

**Authors:** Junbin Lin, Ting Jiang, Xin Zhang, Lu Xia, Yu Gong, Weijing Liao

**Affiliations:** ^1^ Department of Neurological Rehabilitation Zhongnan Hospital of Wuhan University Wuhan China

**Keywords:** extraction, low‐molecular‐weight *Angelica sinensis* polysaccharide, middle cerebral artery occlusion/reperfusion, neurological deficits, neuroplasticity

## Abstract

Neuroplasticity plays a pivotal role in post‐stroke recovery, and *Angelica sinensis* polysaccharide has demonstrated neuroprotective properties. This study investigated the neuroprotective effects of low‐molecular‐weight *Angelica sinensis* polysaccharide (LMW‐ASP) in a rat middle cerebral artery occlusion/reperfusion (MCAO/R) model. Structural characterization revealed that LMW‐ASP (3.9 kDa), containing a pyranose ring and uronic acid, was a heteropolysaccharide that comprised fucose, galactosamine hydrochloride, rhamnose, arabinose, glucosamine hydrochloride, galactose, glucose, xylose, mannose, galacturonic acid, and glucuronic acid (0.007:0.003:0.057:0.209:0.009:0.284:0.303:0.006:0.032:0.083:0.007). Post‐stroke administration of LMW‐ASP (50 mg/kg) significantly improved body weight and neurological functions after MCAO/R. Moreover, microtubule‐associated protein‐2 expression was notably higher in the LMW‐ASP group than in the model group (*p* < 0.01). Furthermore, Golgi‐Cox staining confirmed that LMW‐ASP increased the number of dendritic intersections (*p* < 0.05 for 90–120, 180, and 200 μm; *p* < 0.001 for 70 and 80 μm), the number of terminal branches (*p* < 0.01), the number of branch points (*p* < 0.01), and the density of basal dendritic spines (*p* < 0.05). Finally, transmission electron microscopy revealed an increase in the number of synapses (*p* < 0.001) and presynaptic vesicles (*p* < 0.001) in the LMW‐ASP group. These results suggest that LMW‐ASP is a potential pharmaceutical therapeutic approach for stroke recovery that enhances neuroplasticity.

## Graphical abstract

Low‐molecular‐weight *Angelica sinensis* polysaccharide was extracted, and its characteristics were determined via chromatography and spectroscopy. Our findings demonstrate that LMW‐ASP effectively improves neurological function and neuroplasticity following cerebral ischemia‐reperfusion injury.

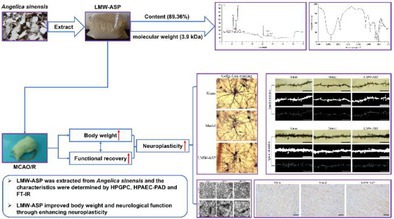

## 1 Introduction

Stroke remains the second leading cause of death worldwide and is the primary contributor to long‐term disability, with ischemic stroke accounting for the majority of cases (approximately 87%) (Fan et al. 2023). Notably, the incidence of ischemic stroke has been on the rise, with 7,804,449 new cases reported in 2021 (Hou et al. 2024). During acute ischemic stroke, there is a drastic decrease in blood flow to the cerebral arteries, resulting in irreversible apoptosis and necrosis of neurons due to hypoxia and ischemia (Cai et al. 2020). Currently, the only pharmacological agent approved by the United States Food and Drug Administration for acute ischemic stroke is tissue plasminogen activator (Fang et al. 2023). However, its use is limited to a small number of patients owing to the risk of hemorrhagic transformation, the narrow therapeutic window, and the challenge of delivering agents to the precise location of the thrombus (Pernal et al. 2020). Consequently, there is an urgent need to develop new and safer therapeutic approaches to treat ischemic stroke.

Neuroplasticity is the capacity of the brain to adaptively alter its structure and function in response to new environments or injuries (Han et al. 2023). It plays a pivotal role in functional restoration following stroke and is involved in many complex processes, such as axonal sprouting, dendritic remodeling, synaptic plasticity, and neurogenesis (Qiao et al. 2023). These processes begin to occur spontaneously within hours after stroke, reach a plateau by 3 to 4 weeks, and have the potential to persist long‐term (F. Yu et al. 2021). In recent years, numerous studies have corroborated the effectiveness of various interventions in improving neuroplasticity after stroke, indicating their potential as therapeutic targets for promoting functional recovery after stroke (Aderinto et al. 2023).

*Angelica sinensis*, which belongs to the family Umbelliferae, is the root of *Angelica sinensis* (Oliv.) Diels. It is one of the most important traditional Chinese medicines and has notable effects on blood enrichment, blood circulation promotion, menstrual cycle regulation, pain alleviation, and bowel relaxation (Chinese Pharmacopoeia Commission 2020). Polysaccharides isolated from various Chinese herbs exhibit multiple pharmacological activities and low toxicity (Zeng et al. 2019). A diverse array of polysaccharides derived from traditional Chinese medicines has been shown to exhibit therapeutic benefits in the treatment of cerebral ischemic injury via multiple mechanisms (Meng et al. 2021; Yuan et al. 2021). *Angelica sinensis* polysaccharide (ASP) is the main water‐soluble component extracted from *Angelica sinensis* and exhibits a range of pharmacological effects, including anti‐inflammatory, anti‐tumor, anti‐oxidative, and the ability to regulate the immune system (Zou et al. 2022). Experimental studies have provided evidence for the neuroprotective role of ASP in cerebral ischemic injury. Ai et al. (2013) reported the antioxidant effects of ASP against cerebral ischemia–reperfusion (I/R) injury. Another study (Xu et al. 2021) demonstrated that ASP significantly alleviates nerve cell apoptosis following cerebral I/R injury by activating the phosphatidylinositol‐3‐kinase/protein kinase B pathway. Additionally, our previous study reported that low‐molecular‐weight ASP (LMW‐ASP) reduced the damage caused by oxidative stress in cerebral cortical neurons and increased the number of microvessels in the brains of stroke‐affected rats (Lei et al. 2014).

Neuroplasticity is a natural process that occurs following a stroke. Certain drugs and rehabilitative interventions facilitate this endogenous neuroplasticity (Szelenberger et al. 2020). This study aimed to extract a type of LMW‐ASP and explore its potential effects on neurological function and neuroplasticity after ischemic stroke.

## 2 Methods

### 2.1 Main Materials and Reagents

The roots of *Angelica sinensis* were purchased from Min County (Gansu Province, China). Standard monosaccharides including fucose (Fuc), galactosamine hydrochloride (GalN), rhamnose (Rha), arabinose (Ara), glucosamine hydrochloride (GlcN), galactose (Gal), glucose (Glc), xylose (Xyl), mannose (Man), fructose (Fru), ribose (Rib), galacturonic acid (GalA), guluronic acid (GluA), glucuronic acid (GlcA), and mannuronic acid (Man A) were supplied by Bo Rui Saccharide Biotech Co. Ltd. (Yangzhou, China). The nylon suture for MACO/R was purchased from Beijing Cinontech Biotechnology Co. Ltd. (Beijing, China). The anti‐microtubule‐associated protein‐2 (MAP‐2) primary antibody was purchased from Boster Biological Technology Co. Ltd. (Wuhan, Hubei, China). An immunohistochemical kit (containing endogenous peroxidase blocking solution, normal animal serum, biotin‐conjugated secondary antibody, and streptavidin‐peroxidase) was purchased from Maixin Technology Co. Ltd. (Fuzhou, Fujian, China). The Hito Golgi‐Cox OptimStain Kit was purchased from Hitobiotec Inc. (Wilmington, DE, USA).

### 2.2 Extraction of ASP

**FIGURE 1:**
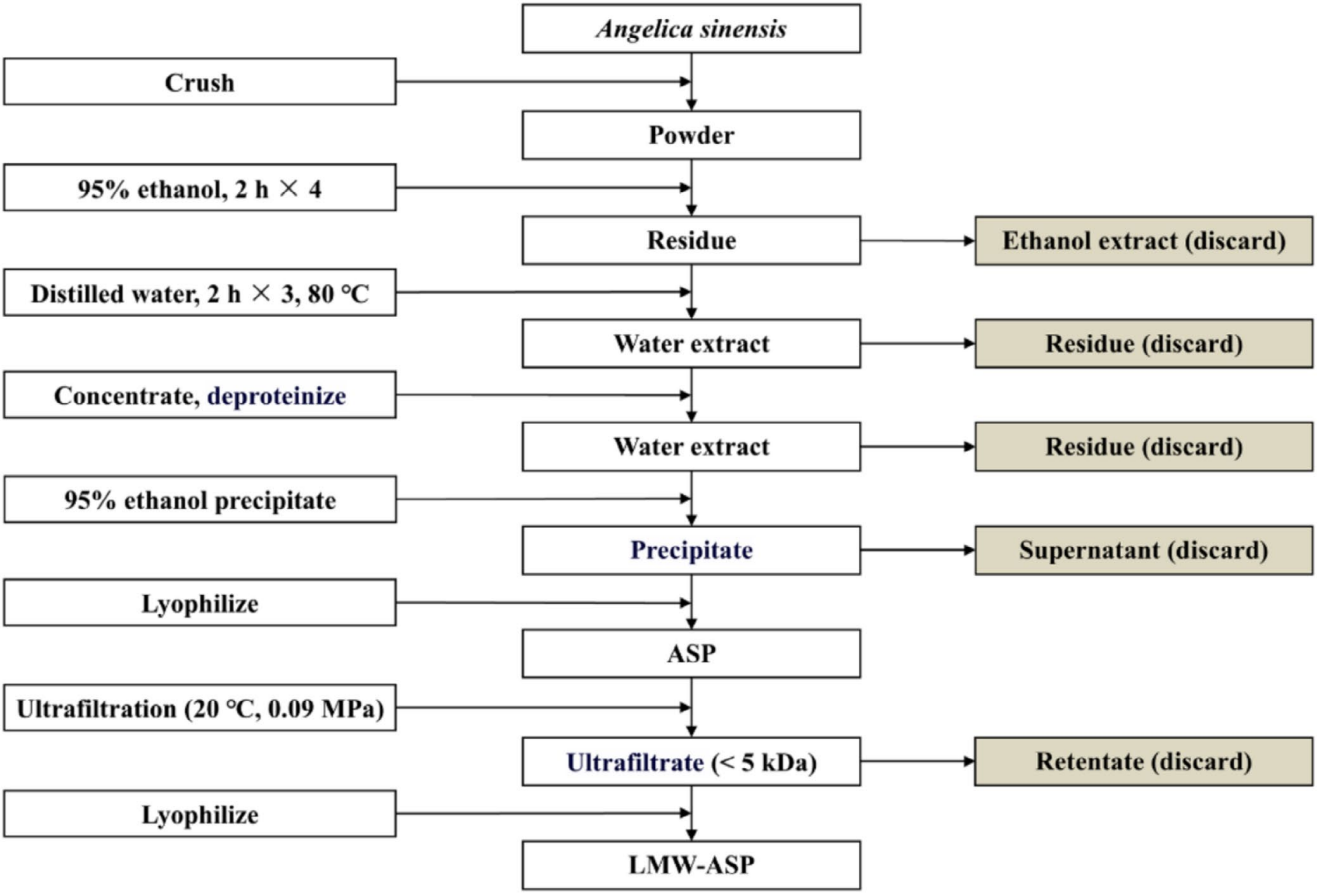
The simple extraction procedure of LMW‐ASP.

After cleaning, the roots of *Angelica sinensis* were thoroughly dried and crushed into a powder. Polysaccharide extraction was performed using an established method of water extraction and ethanol precipitation, as previously described (Tan et al. 2021). To eliminate pigments, monosaccharides, and oligosaccharides, the powder was subjected to four rounds of steeping in 95% ethanol. Subsequently, the residue was extracted three times with distilled water at 80°C. The extracts were concentrated using a vacuum rotary evaporator (RE 52A, Shanghai Yarong Biochemistry Instrument Plant, Shanghai, China), and potential protein contaminants were removed using the Sevag method (Fan and Huang 2023). Next, the solution was precipitated with 5.3 volumes of 95% ethanol and centrifuged at 5000 rpm for 10 min. Finally, the precipitate was lyophilized to yield ASP (Figure 1).

### 2.3 Separation of LMW‐ASP

The ASPs were dissolved in distilled water and separated using an ultrafiltration membrane (Fang et al. 2022). Continuous ultrafiltration was performed through a 5 kDa cut‐off ultrafiltration membrane (Millipore, Bedford, MA, USA) at 20°C and 0.09 MPa. Subsequently, the ultrafiltrate (molecular weight < 5 kDa) was centrifuged and lyophilized to yield LMW‐ASP (Figure 1).

### 2.4 Total Sugar Content of LMW‐ASP

The total sugar content of LMW‐ASP was determined using the phenol‐sulfuric acid method (Bakhshi Jouybari et al. 2023). Briefly, standard glucose solutions ranging from 5 to 50 μg/mL were used to establish a standard curve. Subsequently, a 6% phenol solution and concentrated sulfuric acid were added to both the glucose solutions and the sample (50 μg/mL), and the mixture was kept in hot water for 20 min. Finally, the absorbance was measured at 490 nm.

### 2.5 Molecular Weight of LMW‐ASP

High‐performance gel permeation chromatography (HPGPC) was performed to determine the molecular weight of LMW‐ASP (Guan et al. 2022). The HPGPC system consisted of a BRT105‐103‐101 series gel column (8 × 300 mm) and an RID‐10A refractive index detector (Shimadzu, Kyoto, Japan). The molecular weights of the samples were determined using a standard curve generated from a range of dextran standards.

### 2.6 Monosaccharide Composition Analysis of LMW‐ASP

The monosaccharide composition of LMW‐ASP was measured by high‐performance anion‐exchange chromatography with pulsed amperometric detection (HPAEC‐PAD) according to a previously reported method with minor modifications (Zhang et al. 2022). Briefly, 5 mg of LMW‐ASP was hydrolyzed with 2 mL of trifluoroacetic acid (TFA) at 120°C for 3 h. Subsequently, excess TFA was removed by drying with nitrogen gas. The sample was then dissolved in distilled deionized water and centrifuged at 12,000 rpm for 5 min. Monosaccharides, including Fuc, GalN, Rha, Ara, GlcN, Gal, Glc, Xyl, Man, Fru, Rib, GalA, GluA, GlcA, and Man A, were used as standards.

### 2.7 Fourier Transform Infrared (FT‐IR) Spectroscopy Analysis

Briefly, 2.0 mg of LMW‐ASP was thoroughly blended with 200 mg of potassium bromide and compressed into a pellet. Subsequently, an FT‐IR 650 spectrometer (Tianjin Gangdong Science and Technology Development Co. Ltd., Tianjin, China) was used to identify the functional groups of LMW‐ASP with a scanning wavelength range of 4000~500 cm^−1^ (Liu et al. 2023).

### 2.8 Animals

**FIGURE 2:**
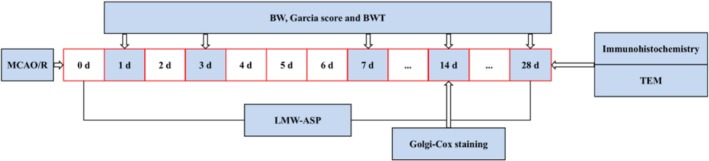
A flow chart of the experimental procedure.

Adult male Sprague–Dawley rats weighing 200–250 g (obtained from the Experimental Animal Center of Wuhan University) were used in this study. The animals were housed in a suitable environment (four animals per cage, 22°C, 60% humidity, and a regular light/dark cycle). All animals were provided with unlimited water and food. Figure 2 presents a concise flowchart illustrating the methodology of the study.

### 2.9 Establishment of the Model

The middle cerebral artery occlusion/reperfusion (MCAO/R) model was established as previously described (Luo et al. 2023). Rats were anesthetized by intraperitoneal injection of pentobarbital sodium (50 mg/kg). Briefly, under anesthesia, a nylon suture was inserted into the right middle cerebral artery through the right common carotid artery and the internal carotid artery. Reperfusion was achieved by gently withdrawing the filaments without anesthesia. All surgical steps were performed in sham‐operated models, except for nylon filament insertion. During the surgical procedure, the rectal temperature was monitored and maintained at 37°C with a heating pad.

### 2.10 Groups

Rats scoring 1–3 points met the inclusion criteria based on a five‐point neurological deficit score (Longa et al. 1989). Rats were randomly assigned to three groups: sham, model, and LMW‐ASP. In the LMW‐ASP group, the rats were treated intraperitoneally with 50 mg/kg LMW‐ASP immediately after reperfusion, and this treatment was continued daily for 28 days. In the sham and model groups, the rats received an equivalent volume of saline.

### 2.11 Body Weight (BW)

The BW of each rat was recorded as a percentage of the baseline BW at 1, 3, 7, 14, and 28 days after MCAO/R.

### 2.12 Neurological Deficit Scores

Neurological function was assessed at 1, 3, 7, 14, and 28 days after MCAO/R using the Garcia score and the beam walking test (BWT). These assessments were conducted by an independent investigator blinded to the treatment allocation. The Garcia score, ranging from 3 points (severe impairment) to 18 points (no impairment), consists of six tests covering spontaneous activity, symmetrical movements of all limbs, forelimbs outstretching, climbing, body proprioception, and response to vibrissal touch (Sun et al. 2023). The BWT was used to assess the coordination and integration of motor movements. During the BWT, the rat traversed a beam (140 × 2.5 × 2.5 cm; 42 cm above the floor) connected to a black box (40 × 20 × 25 cm). The performance of the rats was subjectively assessed according to a previously established method (K. Yu et al. 2013). Before MCAO/R, the rats underwent a three‐day pretraining period.

### 2.13 Immunohistochemistry

At 28 days after MCAO/R, immunohistochemistry (streptavidin‐peroxidase method) (Liu et al. 2019) with some modifications was used to investigate the expression of MAP‐2. Briefly, six rats from each group were deeply anesthetized and transcardially perfused with saline and paraformaldehyde. Brains were immersed overnight in 4% paraformaldehyde. The next day, they were cut into 4‐mm‐thick coronal blocks (bregma: −2 to +2 mm), and 6‐μm‐thick paraffin sections were prepared. The tissue sections were dried, deparaffinized, and rehydrated. Following antigen retrieval by microwave oven heating, an endogenous peroxidase blocking solution was added to the tissue sections for 10 min. The sections were then incubated with normal animal serum, followed by incubation with rabbit anti‐MAP‐2 primary antibody at 4°C overnight. After incubation with a biotin‐conjugated secondary antibody for 15 min, each section was incubated with streptavidin‐peroxidase for another 15 min. Finally, the sections were counterstained with 5% hematoxylin. Images were captured from five non‐overlapping fields at 400× magnification using a microscope (Olympus BX51, Tokyo, Japan), and three random sections from each rat were analyzed. Image‐Pro Plus 6.0 (Media Cybernetics Inc., Bethesda, MD, USA) was used to quantitatively assess the integral optical density (IOD).

### 2.14 Golgi‐Cox Staining

To investigate the changes in dendrites and dendritic spines, a Hito Golgi‐Cox OptimStain Kit was used to stain the neurons. Fourteen days after MCAO/R, six rats from each group were anesthetized, and their brains were removed as soon as possible. Tissues from the forelimb area of the motor cortex (bregma: 0 to +2 mm) were cut into 100‐μm‐thick frozen slices. The entire process of tissue preparation and staining was performed in strict accordance with the manufacturer's instructions and the material safety data sheet. Layer V pyramidal neurons were traced under 400× magnification to analyze dendritic complexity and under 1000× magnification to calculate the density of dendritic spines.

Sholl analysis (Suman et al. 2024), a widely recognized method, was used to quantitatively assess dendritic complexity. Briefly, soma‐centered concentric circles with 10‐μm intervals were delineated. Dendritic complexity was assessed by analyzing the number of dendritic intersections, the total dendritic length, the total number of dendritic branches, the number of terminal branches, and the number of branch points using ImageJ software (National Institutes of Health, Bethesda, MD, USA).

To quantify dendritic spine density, ImageJ software was used to calculate the number of spines and the length of dendritic segments (third‐order branches) for both basal and apical dendrites (Castillo‐Fernández and Silva‐Gómez 2022). Subsequently, the density of dendritic spines was calculated as the number of spines per 1 μm.

### 2.15 Transmission Electron Microscopy (TEM)

At 28 days after MCAO/R, four rats from each group were subjected to TEM analysis. Tissue preparation and sectioning were performed according to methods described in a previous study (J. Lin et al. 2021). Briefly, brain tissues (1 mm^3^) from the peri‐infarct area were subjected to two fixation steps (2.5% glutaraldehyde overnight and 1% OsO4 for 2 h). Following dehydration and embedding, 60‐nm‐thick ultrathin sections were prepared using an ultramicrotome (LKB‐V, LKB Produkter AB, Sweden). The sections were sequentially stained with uranyl acetate and lead citrate for 10 min each. Synapses were quantified using an HT 7700 TEM (Hitachi High‐Tech, Tokyo, Japan) at 5000× magnification. A higher magnification of 10,000× was used to quantify presynaptic vesicles.

### 2.16 Statistical Analysis

The data are expressed as means ± standard deviations. GraphPad Prism 9.5.0 (GraphPad Prism Software Inc., San Diego, CA, USA) was used for statistical analysis. One‐way analysis of variance (ANOVA), followed by Tukey's *post hoc* test, was used to evaluate MAP‐2 expression, total dendritic length, total number of dendritic branches, number of terminal branches, number of branch points, density of dendritic spines, number of synapses, and number of presynaptic vesicles. Repeated‐measures ANOVA, followed by Tukey's *post hoc* test, was used to evaluate body weight and the number of dendritic intersections. Repeated‐measures ANOVA, followed by Sidak's *post hoc* test, was used to evaluate neurological deficit scores. In addition, Pearson's correlation coefficient between the Garcia score and BW was calculated. A *p*‐value < 0.05 was considered statistically significant.

## 3 Results

### 3.1 Total Carbohydrate Content of LMW‐ASP

**FIGURE 3:**
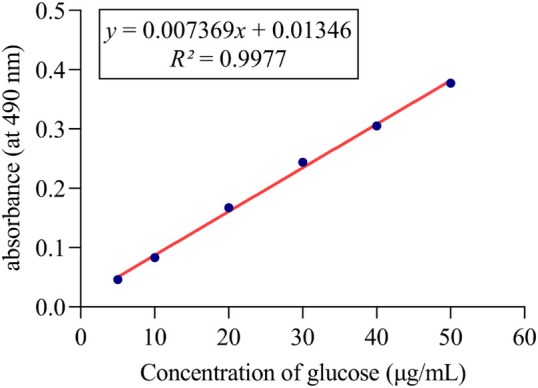
The standard curve of glucose.

The standard linear graph of glucose is shown in Figure 3. The total carbohydrate content of LMW‐ASP was 89.36%.

### 3.2 Molecular Weight of LMW‐ASP

**FIGURE 4:**
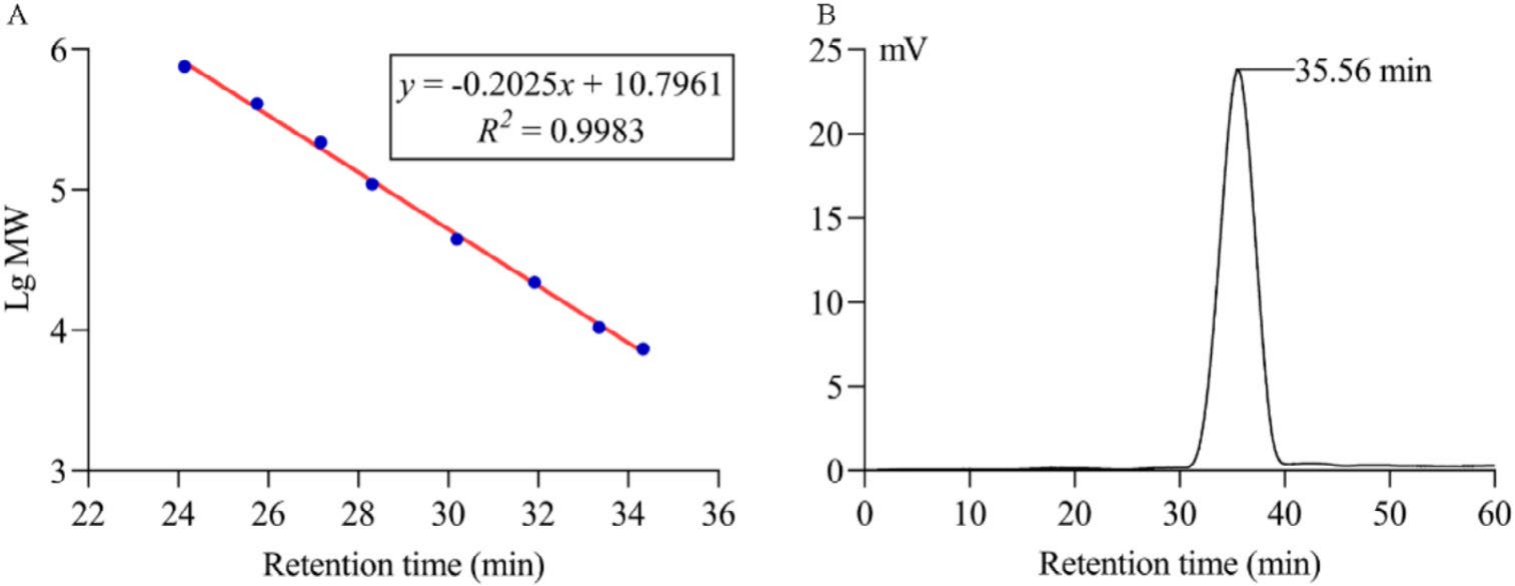
HPGPC analysis of LMW‐ASP. (A) The standard curve of dextran. (B) HPGPC chromatogram profile of LMW‐ASP.

A single and symmetrically sharp peak was observed at 35.56 min in the HPGPC chromatogram (Figure 4B), indicating a homogeneous molecular weight distribution. The average molecular weight of LMW‐ASP was 3.9 kDa based on the regression equation of the standard dextran curve (Figure 4A).

### 3.3 Monosaccharide Composition

**FIGURE 5:**
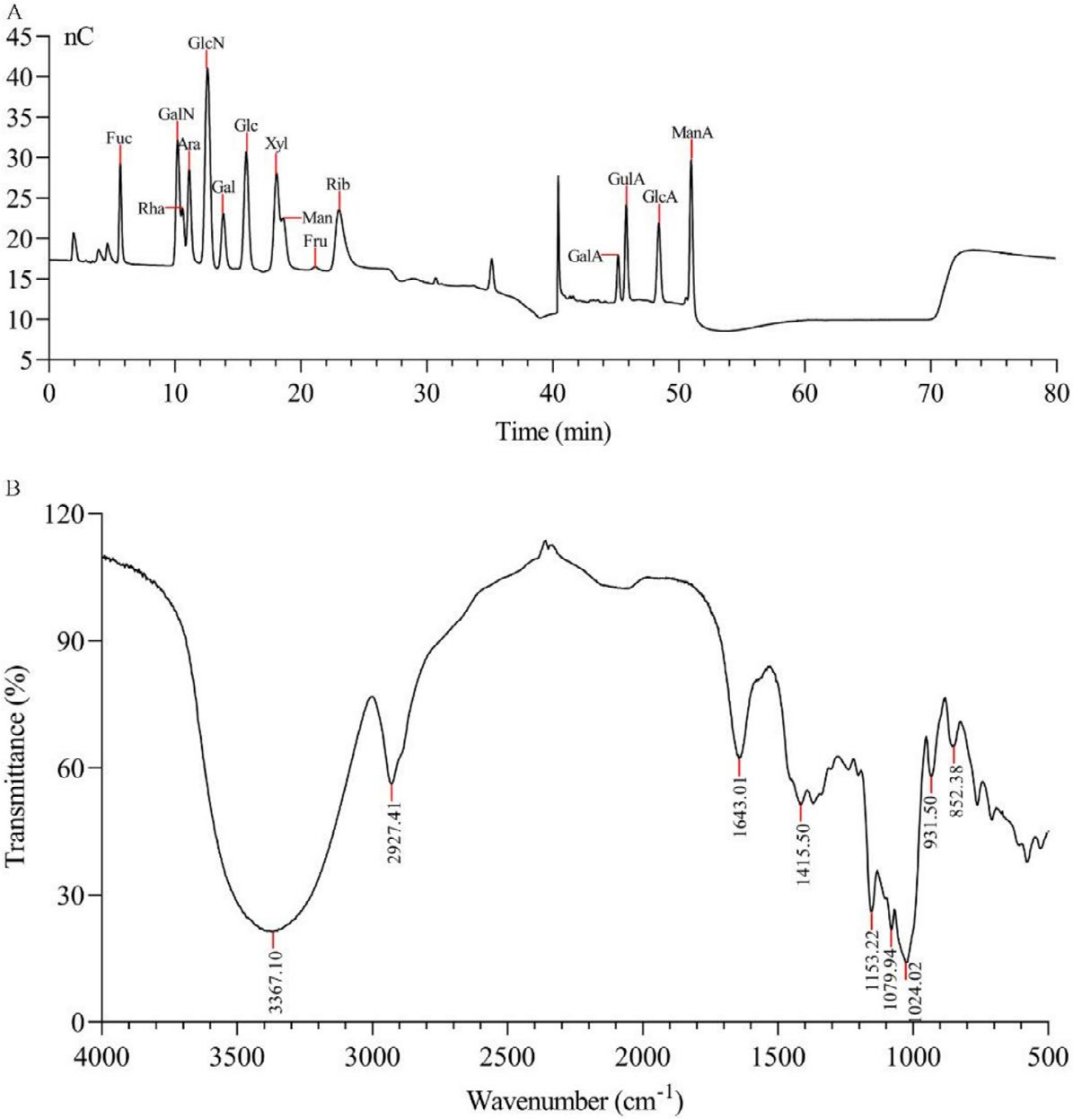
HPAEC‐PAD chromatogram profiles of standard monosaccharide mixed solution (A) and LMW‐ASP (B).

Figure 5A,B shows the results of HPAEC‐PAD for both the mixed monosaccharide standards and LMW‐ASP. The LMW‐ASP was composed of Fuc, GalN, Rha, Ara, GlcN, Gal, Glc, Xyl, Man, GalA, and GlcA. The molar ratio of these monosaccharides was 0.007:0.003:0.057:0.209:0.009:0.284:0.303:0.006:0.032:0.083:0.007 (Table 1).

**TABLE 1:** The monosaccharide composition of LMW‐ASP.

Monosaccharides	Area	Retention time (min)	Molar ratio
Fuc	0.137	5.642	0.007
GalN	0.236	10.2	0.003
Rha	0.765	10.6	0.057
Ara	7.439	11.109	0.209
GlcN	0.982	12.559	0.009
Gal	6.061	13.792	0.284
Glc	15.263	15.617	0.303
Xyl	0.249	18.05	0.006
Man	0.775	18.575	0.032
Fru	0	21.134	0.000
Rib	0	23.009	0.000
GalA	0.994	45.259	0.083
GulA	0	45.817	0.000
GlcA	0.186	48.509	0.007
ManA	0	50.967	0.000

Abbreviations: Ara, arabinose; Fru, fructose; Fuc, fucose; Gal, galactose; GalA, galacturonic acid; GalN, galactosamine hydrochloride; Glc, glucose; GlcA, glucuronic acid; GlcN, glucosamine hydrochloride; GluA, guluronic acid; LMW‐ASP, low‐molecular‐weight *Angelica sinensis* polysaccharide; Man A, mannuronic acid; Man, mannose; Rha, rhamnose; Rib, ribose; Xyl, xylose.

### 3.4 FT‐IR Spectroscopy Analysis

**FIGURE 6:**
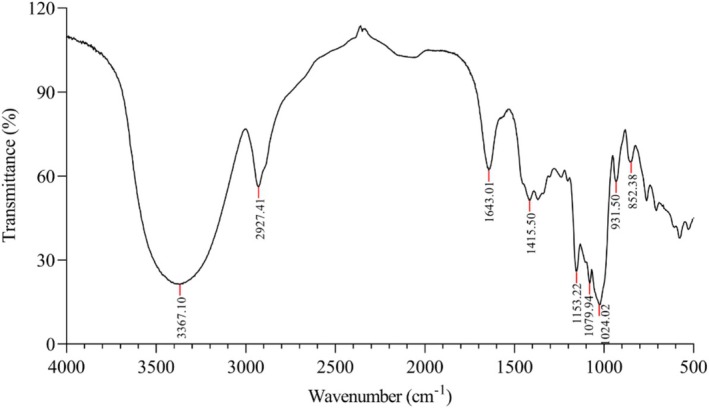
FT‐IR spectrum of LMW‐ASP in the range of 4000–500 cm^−1^.

The FT‐IR spectrum of LMW‐ASP exhibited characteristic polysaccharide absorption peaks, as shown in Figure 6. A prominent and broad peak was observed at 3367 cm^−1^, which was mainly due to the stretching vibrations of the hydroxyl (O–H) groups. The peak at 2927 cm^−1^ was attributed to the stretching vibrations of the alkyl (C–H) groups. The peaks observed at approximately 1643 cm^−1^ and 1416 cm^−1^ were assigned to asymmetrical and symmetrical COO– stretching vibrations, respectively (Jiang et al. 2019; Wu et al. 2021). Additionally, three notable absorption peaks between 1000 and 1200 cm^−1^ were attributed to the stretching and bending vibrations of C–O, C–C, and C–O–C bonds, indicating the possible presence of pyranose rings (Wang et al. 2020). Furthermore, the absorption peaks at 852 and 932 cm^−1^ implied the presence of α‐glycosidic and β‐glycosidic bonds, respectively (Peng et al. 2023).

### 3.5 LMW‐ASP Improved BW in MCAO/R Rats

**FIGURE 7:**
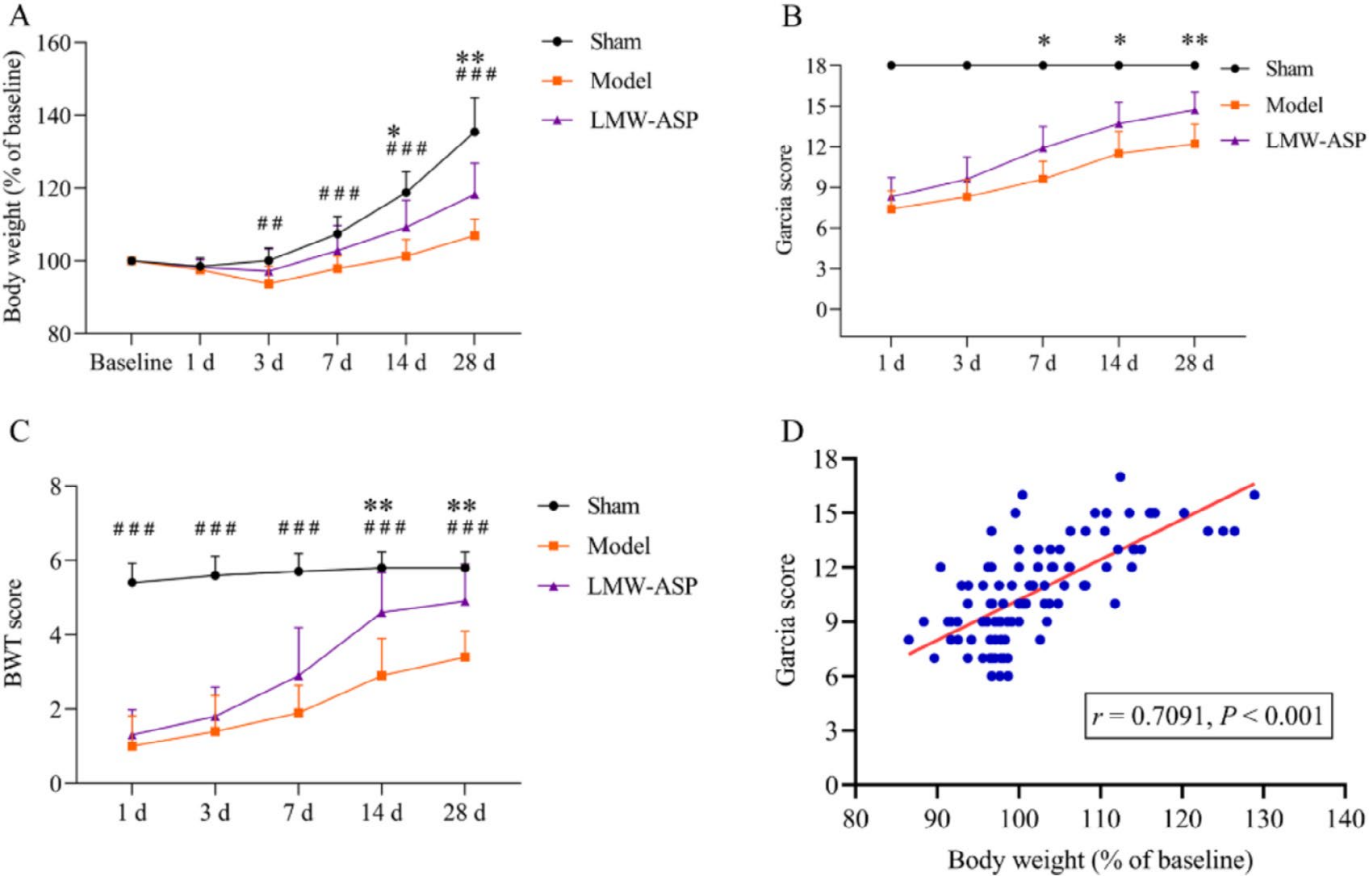
LMW‐ASP improved body weight and neurological deficit scores in MCAO/R rats. (A) BW (recorded as a percentage of the baseline) (mean ± SD, *n* = 10). (B) Garcia score (mean ± SD, *n* = 10). (C) BWT score (mean ± SD, *n* = 10). ## indicates *p* < 0.01, and ### indicates *p* < 0.001 (sham versus model) (AC). * indicates *p* < 0.05, and ** indicates *p* < 0.01 (model versus LMW‐ASP) (AC). D There was a positive correlation between the Garcia score and BW.

As depicted in Figure 7A, BW was recorded as a percentage of the baseline. Generally, BW decreased initially in all three groups of rats and then gradually increased up to 28 days after MCAO/R. Repeated‐measures ANOVA and Tukey's *post hoc* analysis revealed that BW in the model group was significantly lower than that in the sham group from 3 to 28 days following MCAO/R (*p* < 0.01, 3 days; *p* < 0.001, 7, 14, and 28 days). Notably, LMW‐ASP treatment substantially increased BW at 14 and 28 days after MCAO/R compared with the model group (*p* < 0.05, 14 days; *p* < 0.01, 28 days).

### 3.6 LMW‐ASP Ameliorated Neurological Deficits in MCAO/R Rats

The Garcia score and BWT were assessed at 1, 3, 7, 14, and 28 days after MCAO/R. Repeated measures ANOVA and Sidak's *post hoc* analysis revealed no significant difference in the Garcia score between the model and LMW‐ASP groups 1 and 3 days after MCAO/R. However, 7, 14, and 28 days after MCAO/R, the Garcia score in the LMW‐ASP group was significantly higher than that in the model group (*p* < 0.05, 7 and 14 days; *p* < 0.01, 28 days) (Figure 7B). Furthermore, I/R injury resulted in significantly decreased BWT scores compared with the sham group at 1, 3, 7, 14, and 28 days after MCAO/R (all *p <* 0.001), whereas LMW‐ASP‐treated rats showed significant improvements in BWT scores at 14 and 28 days after MCAO/R compared with the model group (all *p <* 0.01) (Figure 7C).

Pearson's correlation analysis revealed a significant positive correlation between the Garcia score and BW after MCAO/R (*r* = 0.7091, *p* < 0.001) (Figure 7D).

### 3.7 LMW‐ASP Upregulated MAP‐2 Expression

**FIGURE 8:**
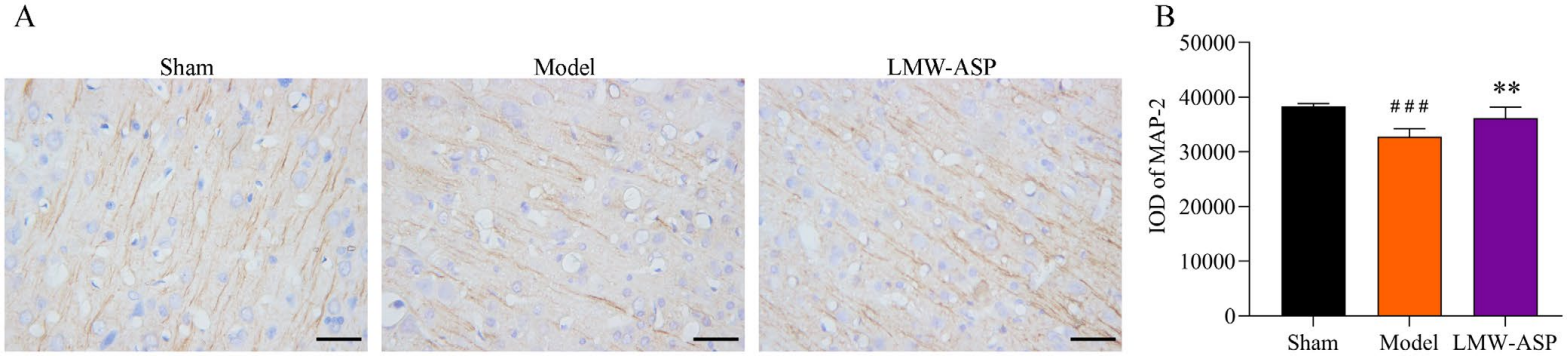
LMW‐ASP upregulated the expression of MAP‐2. (A) MAP‐2 expression was detected by immunohistochemistry (400×, scale bar = 100 μm). (B) The IODs of MAP‐2 (mean ± SD, *n* = 6). ### indicates *p* < 0.001 (sham versus model). ** indicates *p* < 0.01 (model versus LMW‐ASP).

MAP‐2 expression was detected by immunohistochemistry 28 days after MCAO/R (Figure 8A). Fifteen IODs (five fields) were acquired, and the mean value was used for subsequent analysis (*n* = 6 per group). The MAP‐2 expression level was significantly lower in the model group (*p* < 0.001), whereas a notable increase in MAP‐2 expression was observed in the LMW‐ASP group (*p* < 0.01) (Figure 8B).

### 3.8 LMW‐ASP Increased Dendritic Complexity

#### 3.8.1 Number of Dendritic Intersections

**FIGURE 9:**
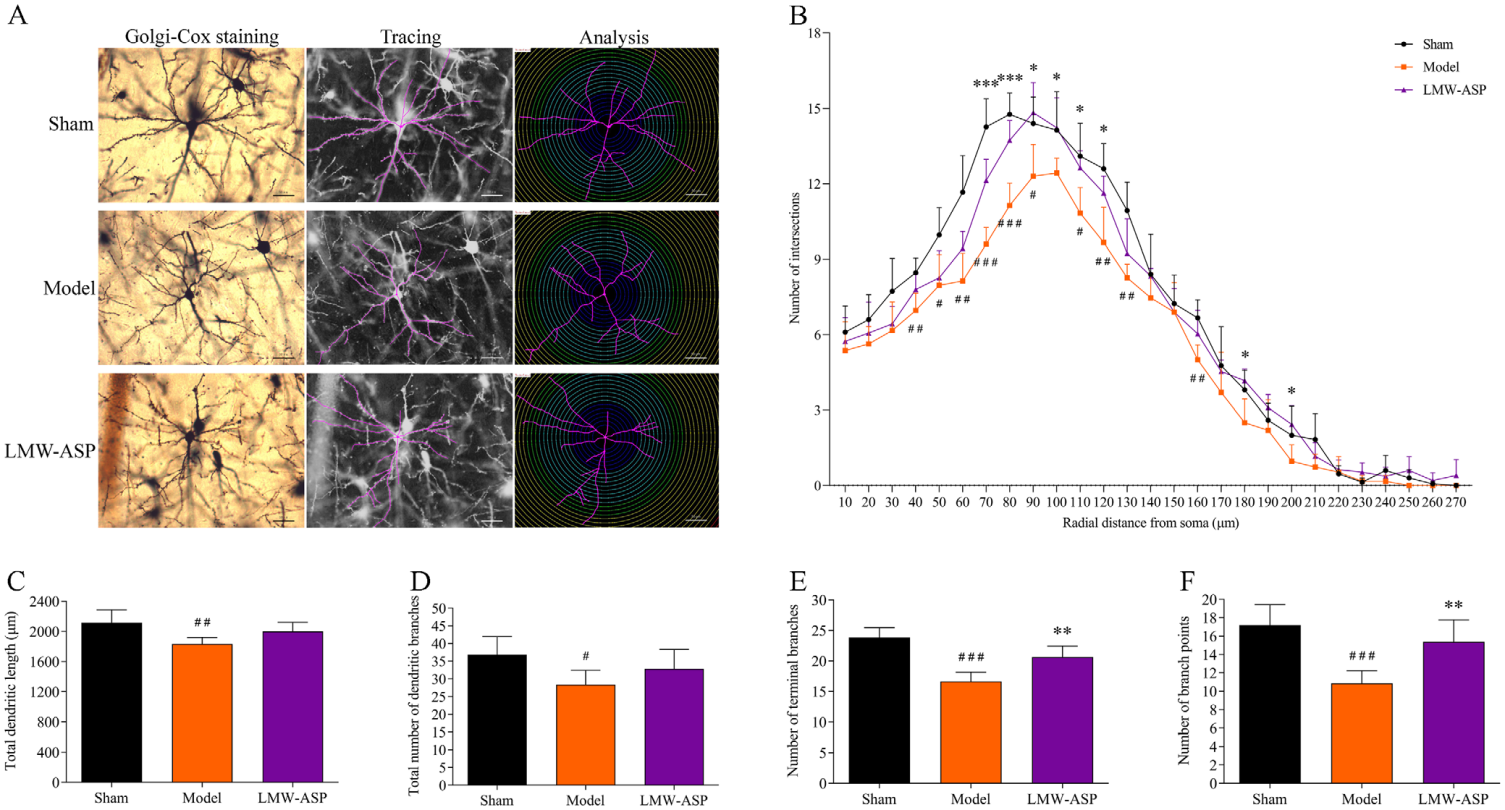
LMW‐ASP improved the dendritic complexity of layer V pyramidal neurons. (A) The dendritic branches of layer V pyramidal neurons were traced and analyzed using the Simple Neurite Tracer plugin in ImageJ software (400×, scale bar = 50 μm). (B) The number of dendritic intersections. (C) The total dendritic length. (D) The total number of dendritic branches. (E) The number of terminal branches. (F) The number of branch points. # indicates *p* < 0.05, ## indicates *p* < 0.01, and ### indicates *p* < 0.001 (sham versus model). * indicates *p* < 0.05, ** indicates *p* < 0.01, and *** indicates *p* < 0.001 (model versus LMW‐ASP).

Golgi‐Cox staining clearly filled the dendritic branches of the layer V pyramidal neurons in the forelimb area of the motor cortex. Dendritic branches were traced using the Simple Neurite Tracer plugin in the ImageJ software (Figure 9A). There was a decrease in the number of branch intersections in layer V pyramidal neurons at 14 days after MCAO/R (compared with the sham group; *p* < 0.05, 50, 90, and 110 μm; *p* < 0.01, 40, 60, 120, 130, and 160 μm; *p* < 0.001, 70 and 80 μm). However, after 2 weeks of LMW‐ASP treatment, there was a significant increase in the number of branch intersections (compared to the model group; *p* < 0.05, 90–120, 180, and 200 μm; *p* < 0.001, 70 and 80 μm) (Figure 9B).

#### 3.8.2 Total Dendritic Length

One‐way ANOVA followed by Tukey's test revealed that the total dendritic length was shorter in the model group than in the sham group (*p* < 0.01). However, LMW‐ASP treatment did not affect dendritic length. The average total dendritic length in LMW‐ASP‐treated rats was 2001.7 ± 119.02 μm, whereas it was 1834.13 ± 87.67 μm in the model group (*p >* 0.05) (Figure 9C).

#### 3.8.3 Total Number of Dendritic Branches

The total number of dendritic branches in the model group was significantly lower than that in the sham group (*p* < 0.05), whereas LMW‐ASP treatment did not have a notable impact on this parameter (*p* > 0.05) (Figure 9D).

#### 3.8.4 Number of Terminal Branches

I/R injury markedly decreased the number of terminal branches (*p* < 0.001). Conversely, the LMW‐ASP group exhibited an increase in the number of terminal branches (*p* < 0.01) (Figure 9E).

#### 3.8.5 Number of Branch Points

The number of branch points in the model group was significantly lower than that in the sham group (*p* < 0.001). However, the number of branch points in the LMW‐ASP‐treated rats was significantly greater than that in the model group (*p* < 0.01) (Figure 9F).

### 3.9 LMW‐ASP Increased the Density of Dendritic Spines

**FIGURE 10:**
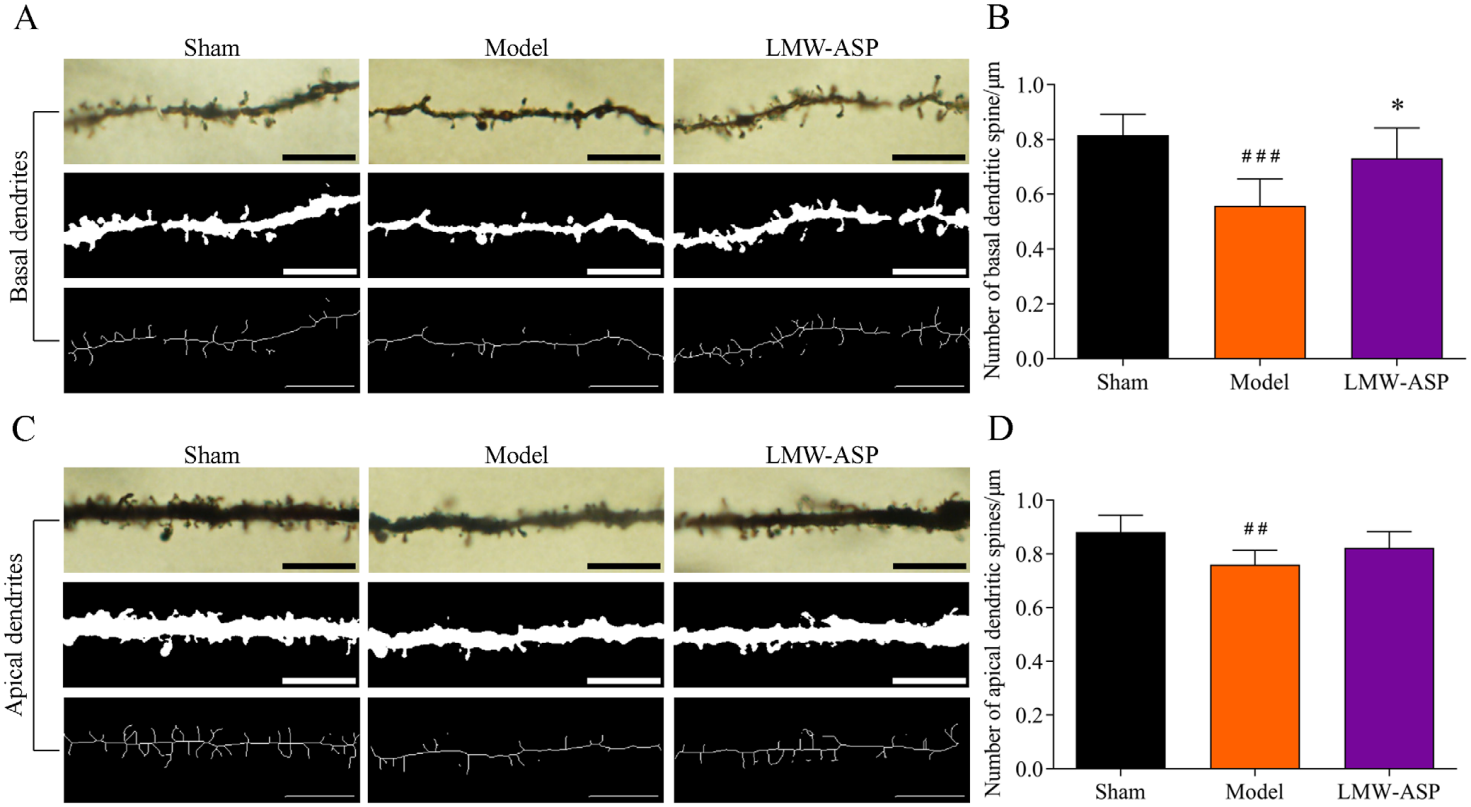
LMW‐ASP increased the density of dendritic spines in layer V pyramidal neurons. (A) Representative images of basal dendritic spines (1000×, scale bar = 10 μm). (B) The density of basal dendritic spines was expressed as the number of dendritic spines/μm (mean ± SD, *n* = 6). (C) Representative images of apical dendritic spines (1000×, scale bar = 10 μm). (D) The density of apical dendritic spines was expressed as the number of dendritic spines/μm (mean ± SD, *n* = 6). ## indicates *p* < 0.01, and ### indicates *p* < 0.001 (sham versus model). * indicates *p* < 0.05 (model versus LMW‐ASP).

Dendritic spines of layer V pyramidal neurons were distinctly delineated using Golgi‐Cox staining (Figure 10A,C). Fourteen days after MCAO/R, I/R injury resulted in a significant reduction in dendritic spine density in both the basal (*p* < 0.001) and apical (*p* < 0.01) dendrites compared to that in the sham group. LMW‐ASP treatment notably increased the density of basal dendritic spines (*p* < 0.05), whereas there was no significant difference in apical dendritic spine density between the model and LMW‐ASP groups (Figure 10B,D).

### 3.10 LMW‐ASP Increased the Number of Synapses and Presynaptic Vesicles

**FIGURE 11:**
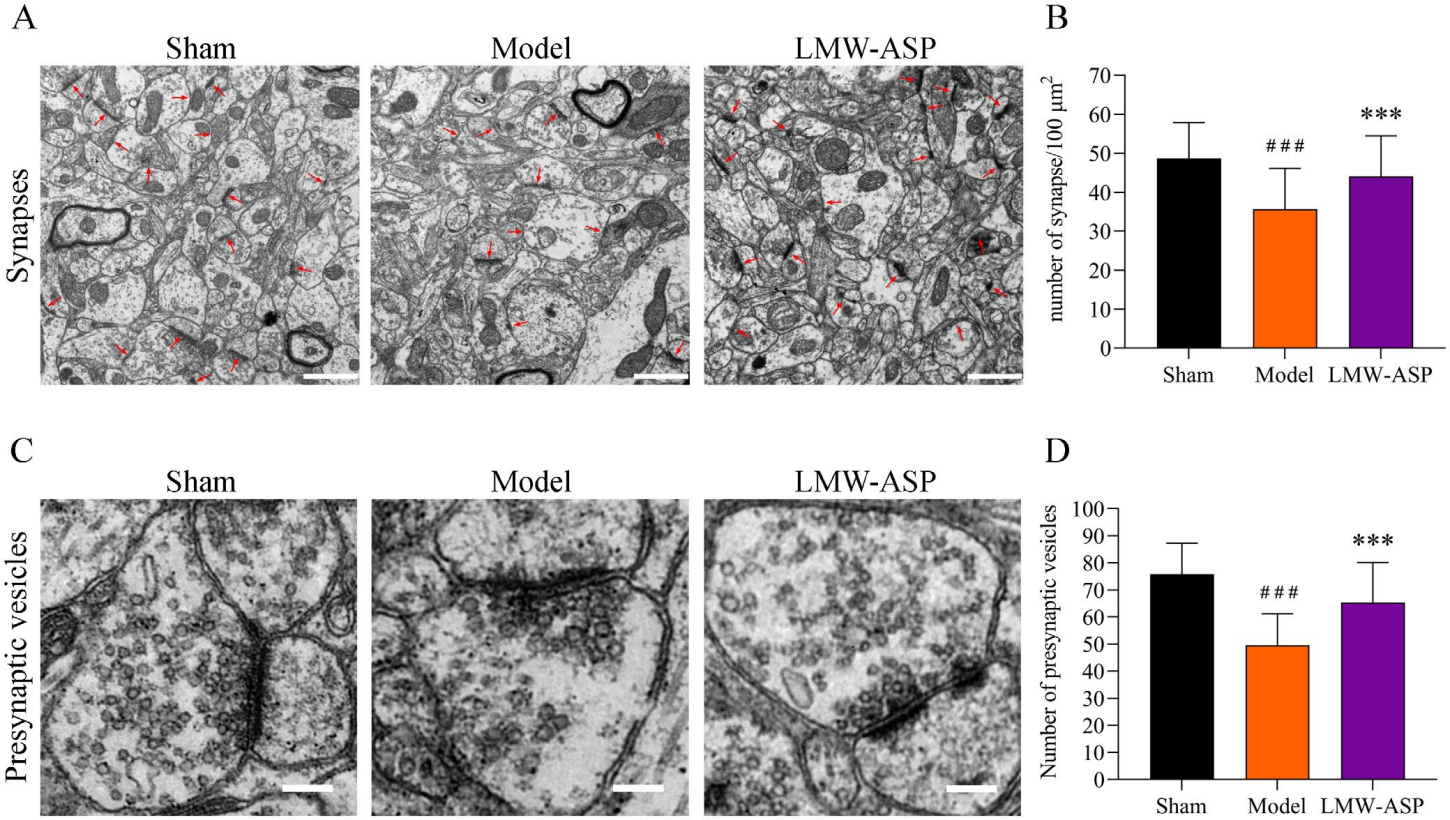
LMW‐ASP increased the number of synapses and presynaptic vesicles. (A) Representative TEM images of synapses in the peri‐infarct area (red arrows represent synapses; 5000×, scale bar = 1 μm). (B) The density of synapses. (C) Representative TEM images of presynaptic vesicles in the peri‐infarct area (10,000×, scale bar = 200 nm). (D) The number of presynaptic vesicles. ### indicates *p* < 0.001 (sham versus model). *** indicates *p* < 0.001 (model versus LMW‐ASP).

Forty electron microscope fields at 5000× magnification were randomly selected from each experimental group for synapse quantification. As shown in Figure 11A, the synapses were distinctly discernible using TEM. I/R caused a significant decrease in synaptic density compared to that in the model group (*p* < 0.001), which was partially reversed by the administration of LMW‐ASP (*p* < 0.001) (Figure 11B). The number of presynaptic vesicles in each group was counted at 10000× magnification using 40 electron microscope fields (Figure 11C). There was a significant decrease in the number of presynaptic vesicles in the model group (*p* < 0.001), whereas the LMW‐ASP group exhibited a pronounced increase (*p* < 0.001) (Figure 11D).

## 4 Discussion

Polysaccharides, Z‐ligustilide, and ferulic acid are the major bioactive components of *Angelica sinensis* and have been demonstrated to mitigate ischemic damage in experimental rodent models of cerebral ischemia via diverse mechanisms (Cheng et al. 2020). Crude ASP has several drawbacks, such as low stability, fast metabolism, and reduced reproducibility, resulting from its high molecular weight and complex purification process. Several studies have suggested that polysaccharides with high molecular weights exhibit high intrinsic viscosity, low water solubility, and low bioactivity (Ma et al. 2020). In this study, we extracted a type of LMW‐ASP (89.36%) from *Angelica sinensis* using water extraction and ethanol precipitation, followed by ultrafiltration. This polysaccharide was creamy white in color and highly soluble in water. HPGPC analysis revealed that LMW‐ASP displayed a single, symmetrical, and narrow peak, indicating a homogeneous molecular weight distribution. Moreover, the average molecular weight of LMW‐ASP was determined to be 3.9 kDa, which is notably lower than that of most previously reported ASPs (Nai et al. 2021). In our previous study, we isolated LMW‐ASP (molecular weight < 20 kDa) and demonstrated its ability to enhance antioxidant activity in cerebral cortical neurons, increase microvessel density, and improve blood flow following ischemia (Lei et al. 2014). In this study, LMW‐ASP, with its higher purity and lower molecular weight, may exhibit even more pronounced bioactive properties.

We also analyzed the monosaccharide composition of LMW‐ASP using HPAEC‐PAD. The analysis revealed that the monosaccharide constituents of ASP included Fuc, GalN, Rha, Ara, GlcN, Gal, Glc, Xyl, Man, GalA, and GlcA, with molar ratios of 0.007:0.003:0.057:0.209:0.009:0.284:0.303:0.006:0.032:0.083:0.007, respectively. In the present study, Ara, Gal, and Glc were found to be the main components. These results confirm that ASP is a heteropolysaccharide, which is consistent with the findings of previous studies (Nai et al. 2021).

FT‐IR is a practical qualitative analysis tool for determining the structure of polysaccharides. Figure 6 shows a strong and wide band at 3367 cm^−1^ for O–H stretching vibrations and a relatively weak absorption peak at 2927 cm^−1^ for C–H stretching vibrations, which are characteristic groups of carbohydrates (Zhou et al. 2022). The peaks at 1641 and 1415 cm^−1^ suggest the presence of uronic acid in LMW‐ASP, which is consistent with the analysis of its monosaccharide composition. Moreover, the absorption peaks below 1200 cm^−1^ suggest the existence of pyranose rings, α‐glycosidic bonds, and β‐glycosidic bonds. These results show almost no differences when compared to the FT‐IR signals of other ASPs (S. Zhang et al. 2010; Zou et al. 2022).

A few studies have indicated that plant‐derived polysaccharides may offer protective effects against central nervous system diseases by modulating neuroplasticity (Lin et al. 2014; Ming et al. 2023; Xie et al. 2024). The present study aimed to elucidate the potential protective role of LMW‐ASP against cerebral ischemic injury and to assess its positive impact on neuroplasticity.

The BW data in our study revealed significant differences between the sham and model groups at all‐time points, except one day after MCAO/R. This observation suggests that MCAO/R induced a relatively severe decrease in BW. Over time, we observed a characteristic fall‐and‐rise pattern in BW, which is consistent with the findings of previous research (C. Li et al. 2022; Pan et al. 2021). Changes in BW may serve as a promising surrogate for assessing general health and could potentially be a supplementary outcome parameter after ischemic cerebral injury (Michalski et al. 2012). Our findings indicate that the administration of LMW‐ASP led to improvements in both functional recovery and BW after MCAO/R. These results suggest that LMW‐ASP has the potential to enhance the general health status of rats subjected to MCAO/R. Subsequently, we performed Pearson's correlation analysis and detected a significant positive correlation between the Garcia score and BW following MCAO/R. This finding indicates the potential of BW to serve as a valuable outcome indicator in stroke‐related studies.

Neuroplasticity, which encapsulates the brain's ability to adapt to and modify the structure and function of neurons and their intricate networks in response to stroke‐induced damage, serves as the fundamental basis for functional recovery after stroke (Li 2017). Synaptogenesis and synaptic reconstruction play pivotal roles in neuroplasticity. Some synaptic changes, including changes in the number of synapses and the density and morphology of dendritic spines, are associated with neuroplasticity (Li et al. 2020). MAP‐2 is a protein primarily expressed in neuronal dendrites and is critical for microtubule assembly, stabilization, and cross‐linking (Gumy et al. 2017). MAP‐2 is recognized as a specific marker of axon and dendrite regeneration (Zhao et al. 2021). We analyzed the changes in MAP‐2 expression and discovered that LMW‐ASP significantly upregulated the expression at 28 days after stroke. These findings suggest that LMW‐ASP induces dendritic modifications at the protein level, thereby contributing to neuroplasticity and functional recovery.

Synaptic plasticity and functional remapping are crucial for functional recovery after stroke (Joy and Carmichael 2021). Dendritic spines, which are minuscule membrane specializations that form the postsynaptic components of most excitatory synapses, play an indispensable role in the regulation of synaptic transmission (Zagrebelsky et al. 2020). Dendrites and dendritic spines are particularly vulnerable to damage after MCAO/R (Xie et al. 2019). Accumulating evidence indicates that dendritic remodeling with structural changes occurs after ischemia and can be enhanced by drugs or rehabilitative training after experimental stroke (Hu et al. 2020). In this study, Golgi‐Cox staining was used to elucidate the dendritic plasticity of layer V pyramidal cells within the forelimb area. Our findings revealed that MCAO/R decreased dendritic complexity and dendritic spine density. However, LMW‐ASP partially restored dendritic complexity and increased the density of basal dendritic spines, which was consistent with the observed MAP‐2 levels. Synaptic vesicles are important sites for storing neurotransmitters and are responsible for releasing their contents into the synaptic cleft (Binotti et al. 2021). The number of synaptic vesicles is usually positively correlated with the number of released neurotransmitters (Tang et al. 2023). The number of synapses and presynaptic vesicles was significantly decreased in the model group, indicating a decline in synaptic transmission. Notably, LMW‐ASP attenuated this reduction in synaptic density and the number of presynaptic vesicles in the peri‐infarct area. These results suggest that enhanced neuroplasticity may be a key mechanism underlying the therapeutic effects of LMW‐ASP in the treatment of ischemic stroke.

However, this study has several limitations. First, the specific mechanism by which LMW‐ASP regulates neuroplasticity is still unclear. A representative neurotrophin, brain‐derived neurotrophic factor (BDNF), may be one of the potential mechanisms involved. BDNF plays a key role in regulating spine formation, synaptic connections, synapse structure, neurotransmitter release, and plasticity at synaptic junctions (Yang et al. 2023). A previous study (Du et al. 2020) revealed that ASP markedly upregulated the expression level of BDNF and its specific receptor tropomyosin‐related kinase B in Alzheimer's disease rats. This effect may represent a mechanism through which LMW‐ASP contributes to the enhancement of neuroplasticity, and further research is needed to confirm this hypothesis. Second, this study did not include a comparison of LMW‐ASP with analytical standards or positive controls. The incorporation of analytical standards or positive controls in future research may help to obtain more accurate conclusions.

## 5 Conclusion

LMW‐ASP was obtained from *Angelica sinensis* after extraction and separation. The total carbohydrate content was 89.36%, and the molecular weight was approximately 3.9 kDa. The results of HPAEC‐PAD analysis indicated that LMW‐ASP was a heteropolysaccharide composed of Fuc, GalN, Rha, Ara, GlcN, Gal, Glc, Xyl, Man, GalA, and GlcA (0.007:0.003:0.057:0.209:0.009:0.284:0.303:0.006:0.032:0.083:0.007). Our study revealed that LMW‐ASP ameliorated neurological deficits following cerebral I/R injury. Moreover, LMW‐ASP promoted dendritic complexity and increased the density of dendritic spines. Additionally, TEM revealed that LMW‐ASP increased the number of synapses and presynaptic vesicles. In summary, LMW‐ASP may be a potential therapeutic pharmaceutical approach that could effectively ameliorate neurological deficits following stroke by improving neuroplasticity.

## Author Contributions

**Junbin Lin:** funding acquisition (equal), investigation (equal), methodology (equal), project administration (equal), writing – original draft (equal). **Ting Jiang:** investigation (equal), methodology (equal), project administration (equal), writing – original draft (equal). **Xin Zhang:** conceptualization (equal), data curation (lead), formal analysis (lead), supervision (equal), writing – review and editing (equal). **Lu Xia:** resources (equal), software (lead), validation (equal). **Yu Gong:** resources (equal), validation (lead), visualization (equal). **Weijing Liao:** conceptualization (equal), funding acquisition (equal), supervision (equal), writing – review and editing (equal).

## Ethics Statement

All experimental procedures were approved by the Institutional Animal Care and Use Committee of the Wuhan University Center of Animal Experiments (approval number: 2012040). The experiments were conducted in accordance with the Animal Research: Reporting of in vivo Experiments (ARRIVE) guidelines for the care and use of research animals.

## Conflicts of Interest

The authors declare no conflicts of interest.

## Data Availability

The data that support the findings of this study are available from the corresponding author upon reasonable request.
